# Pre-referral intranasal artesunate powder for cerebral malaria: a proof-of-concept study

**DOI:** 10.1186/s12936-022-04309-0

**Published:** 2022-10-11

**Authors:** Yobouet Ines Kouakou, Aurelien Millet, Elodie Fromentin, Nathalie Hauchard, Gonçalo Farias, Maxime Fieux, Aurelie Coudert, Roukayatou Omorou, Ibrahim Bin Sa’id, Adeline Lavoignat, Guillaume Bonnot, Anne-Lise Bienvenu, Stephane Picot

**Affiliations:** 1 Univ Lyon, Malaria Research Unit, SMITh, ICBMS, UMR 5246 CNRS-INSA-CPE-University Lyon1, 69622 Villeurbanne, France; 2 Groupement Hospitalier Nord, Institut de Parasitologie et Mycologie Médicale, Hospices Civils de Lyon, 69004 Lyon, France; 3 Laboratory of Biochemistry and Pharmacotoxicology, Lyon-Sud Hospital, Hospices civils de Lyon, 69310 Pierre-Bénite, France; 4 Univ Lyon, CCSM, ICBMS, UMR 5246 CNRS-INSA-CPE-University Lyon1, 69622 Villeurbanne, France; 5 Aptar Pharma, Le Vaudreuil, France; 6 Hospices Civils de Lyon, Centre Hospitalier Lyon Sud, Service d’ORL, d’otoneurochirurgie et de chirurgie cervico-faciale, 69310 Pierre Bénite, France; 7 Department of Pediatric Otolaryngology—Head & Neck Surgery, Femme Mere Enfant Hospital—Hospices Civils de Lyon, 69008 Lyon, France; 8 Service Pharmacie, Groupement Hospitalier Nord, Hospices Civils de Lyon, 69004 Lyon, France

**Keywords:** Severe malaria, Artesunate, Pre-referral treatment, Nose-to-brain delivery, Nasal mucosa, Nasal cast

## Abstract

**Background:**

Malaria still kills young children in rural endemic areas because early treatment is not available. Thus, the World Health Organization recommends the administration of artesunate suppositories as pre-referral treatment before transportation to the hospital in case of severe symptoms with an unavailable parenteral and oral treatment. However, negative cultural perception of the rectal route, and limited access to artesunate suppositories, could limit the use of artesunate suppositories. There is, therefore, a need for an alternative route for malaria pre-referral treatment. The aim of this study was to assess the potential of intranasal route for malaria pre-referral treatment.

**Methods:**

The permeability of artesunate through human nasal mucosa was tested in vitro. The Transepithelial Electrical Resistance (TEER) of the nasal mucosa was followed during the permeation tests. Beside, regional deposition of artesunate powder was assessed with an unidose drug delivery device in each nostril of a nasal cast. Artesunate quantification was performed using Liquid Chromatography coupled to tandem Mass Spectrometry.

**Results:**

The experimental model of human nasal mucosa was successfully implemented. Using this model, artesunate powder showed a much better passage rate through human nasal mucosa than solution (26.8 ± 6.6% versus 2.1 ± 0.3%). More than half (62.3%) of the artesunate dose sprayed in the nostrils of the nasal cast was recovered in the olfactory areas (44.7 ± 8.6%) and turbinates (17.6 ± 3.3%) allowing nose-to-brain and systemic drug diffusion, respectively.

**Conclusion:**

Artesunate powder showed a good permeation efficiency on human nasal mucosa. Moreover it can be efficiently sprayed in the nostrils using unidose device to reach the olfactory area leading to a fast nose-to-brain delivery as well as a systemic effect. Taken together, those results are part of the proof-of-concept for the use of intranasal artesunate as a malaria pre-referral treatment.

## Background

Malaria is one of the deadliest human infectious diseases, responsible for more than 600,000 deaths in 2021, most of them being young children in sub-Saharan Africa [1, 2]. Whereas a great majority of cases are uncomplicated and successfully treated with oral anti-malarials, severe malaria may occur in non-immune patients, including children under five years old living in high transmission endemic regions [1, 3–5]. A rapid and optimal anti-malarial treatment is critical to improve the outcome of severe cases. The first-line treatment recommended for severe malaria is intravenous artesunate, a semisynthetic derivative of artemisinin. In rural areas where intravenous treatments are not available, the World Health Organization (WHO) recommends the use of a pre-referral artesunate treatment before addressing the patient to the nearest hospital for appropriate care [1, 6]. Pre-referral artesunate treatment is a single dose of artesunate administered intrarectally to young children (< 6 years old) at risk of severe malaria when oral route is unavailable [1, 6]. This pre-referral artesunate treatment using suppositories was demonstrated to reduce the risk of death or permanent disability [6]. Pharmacokinetic studies showed large interindividual variability with a bioavailability of 11.7 to 54.4% (mean = 25.6%), but a 12-h parasite reduction ratio comparable to intravenous artesunate [7]. Among its advantages, the rectal route is non-invasive, usable in unconscious or vomiting patients, allows systemic drug absorption, and avoids at least partially the hepatic first-pass effect [8].

However, a negative cultural perception of the rectal route, as well as reports of melting artesunate suppositories under tropical and subtropical temperatures, and the possible expulsion of the suppositories could limit its use [8–10]. Although two artesunate suppositories have now been prequalified by the WHO, there is still little evidence for the implementation of this pre-referral treatment into endemic countries’ guidelines for severe malaria management [11, 12]. Consequently, the availability of rectal AS is reduced as observed in Ethiopia [13] and Kenya according to a recent national survey of primary public health facilities conducted between 2017 and 2021 [14]. In this context, there is a need for the development of an alternative artesunate pre-referral treatment.

The nasal route was described as an alternative to parenteral and oral routes [15]. It is better accepted than the rectal route and it avoids the first-pass hepatic metabolism. It allows to bypass the blood-brain barrier (BBB) and facilitates the diffusion of drugs directly inside the brain microvasculature constituted by endothelial cells held together by tight junctions leading to continuous and non-fenestrated vessels. This BBB restricts considerably the diffusion of molecules between blood and central nervous system (CNS) [16]. However, rapid alteration of the BBB is associated with a broad spectrum of diseases including cerebral malaria (CM). During CM, the neurovascular unit (NVU), defined as the interaction between of the microvasculature (endothelial and pericyte cells) and neural cells (neurons, astrocyte, microglia) is severely impacted by the local sequestration of infected red blood cells, leading to a neuroinflammatory process and BBB breakdown [17]. This process resulting in neurotoxicity and axonal injury associated with a compromised blood-nerve barrier [18, 19] and perivascular micro-haemorrhages may enable substance to cross the BBB by passive diffusion in both directions [20]. Taken together, these pathological events argued for the potential benefit of artesunate immediate access to the NVU during CM, providing a rapid reduction of the parasite burden and the resultant local inflammatory process. Indeed, the advantages of the nasal route include non-invasiveness, ease of drug administration, and fast drug absorption [15, 21]. The richly vascularized nasal mucosa is effective for drug absorption depending on the regional nasal deposition pattern [22]. The respiratory and the olfactory mucosa are of particular interest for drug absorption: the respiratory mucosa is the preferential site for systemic drug absorption [21, 23, 24], whereas the olfactory mucosa is the preferential site for nose-to-brain drug absorption [21, 24]. The olfactory zone provides a direct access to the brain through the olfactory and trigeminal nerve termination in the cavity. Nose-to-brain drug delivery use the endoneurial microvessels within the nerve fascicle and the perineurium [25, 26] to allow the passage of substances into the brain within minutes. Thus, nasal route allows to bypass the BBB that is of utmost importance in the case of cerebral malaria characterized by sequestration of infected red-blood cells in cerebral capillaries.

Among nasal route shortcomings, are the short retention time of drugs because of mucociliary clearance, metabolic degradation in the nasal cavity, and restricted dosing volume, mostly in children [21, 27, 28]. Over the last two decades, the nasal route was actively assessed for the delivery of various drugs, including vaccines, hormones (insulin, melatonin), opioids, and triptans [15, 29–32]. Nasal administration of sumatriptan powder is now recommended by the Food and Drug Administration (FDA) for the treatment of acute migraines in adults [33]. Compared to the oral route, the nasal route is associated with faster and greater relief of migraines symptoms [34]. Intranasal administration of artesunate for severe malaria treatment would allow both the systemic effects of artesunate, and the nose-to-brain delivery of the drug that would prevent cerebral malaria. This statement is based on preliminarydata obtained in a murine model of cerebral malaria that demonstrated the nasal route to prevent the development of complications including cerebral malaria [9]. It was demonstrated that dihydroartemisinin, the main metabolite of artesunate, was recovered into the brain of mice after artesunate intranasal treatment and that early artesunate treatment using intranasal route prevented the development of cerebral malaria.

In this context, there is evidence to further assess the nasal route for artesunate. The objectives of this study were, firstly, to assess artesunate permeation and cytotoxicity using a model of human nasal mucosa and, secondly, to study the nasal deposition pattern of the drug sprayed in a human nasal cast model. This would contribute to the establishment of the nasal route as an alternative for pre-referral treatment of malaria.

### Methods

#### Chemicals, culture media, and drug solutions

Artesunate (Chemical Abstract Service CAS number: 88495-63-0; IPCA #19003AS6RII [1 kg]) was purchased from IPCA Laboratories Limited (Mumbai, India) through Hepartex (Saint-Cloud, France). Krebs-ringer buffer (KRB) was prepared by dissolving 6.8 g NaCl, 0.4 g KCl, 0.14 g NaH_2_PO_4_**ּ** H_2_O, 2.1 g NaHCO_3_, 3.575 g HEPES, 1.0 g D-glucose, 0.2 g MgSO_4_ּ 7H_2_O, and 0.26 g CaCl_2_ּ 2H_2_O in 1 L of distilled sterile water. HEPES, D-glucose, and NaHCO_3,_ were purchased from Carl Roth GmbH (Karlsruhe, Germany). All other KRB reagents, 0.25% (w/v) trypsin-EDTA, Trypan blue stain (0.4%), phosphate buffer solution (PBS) tablets, fluorescein sodium salt (NaF), 3-(4,5-dimethylthiazol-2-yl)-2,5-diphenyltetrazolium bromide (MTT), and Dimethyl sulfoxide (DMSO), were purchased from Sigma-Aldrich Merck (Saint-Louis, Missouri, USA). Culture medium included minimum essential medium (MEM), 10% (v/v) heat-inactivated sterile foetal bovine serum (FBS), 1% (w/v) L-Glutamine, 1% (v/v) non-essential amino-acids (NEAA), and 20 µg/ml gentamicin. MEM, heat-inactivated FBS, L-glutamine, and NEAA, were purchased from Sigma-Aldrich Merck (Saint-Louis, Missouri, USA). Nasal epithelial cells RPMI 2650 (RMPI 2650 ECACC 88,031,602) were purchased from the European Collection of Authenticated Cell Cultures (ECACC, Porton, Wiltshire, England). Falcon® Cell-culture flasks, Flacon® 96-well plates and Corning Costar® 12-well plates were purchased from Corning (Glendale, Arizona, USA). ThinCert® tissue-culture inserts for 12-Well plates (polyethylene terephthalate membrane, 1.13 cm², 0.4 mm pore size) were purchased from Greiner Bio-One (Kremsmünster, Austria). Artesunate and artesunate-d4 standards for mass spectrometry (MS) analysis were purchased from Alsachim (Illkirch-Graffenstaden, France). Ultrapure water was obtained from Biosolve (Dieuze, France) and Thermo Fischer Scientific (Massachusetts, USA). Mass spectrometry-grade (MS-grade) acetonitrile was obtained from Biosolve (Dieuze, France). MS-grade ammonium acetate, acetic acid and methanol were obtained from (Thermo Fischer Scientific (Massachusetts, USA). Formic acid and ammonium formiate were purchased from Sigma Aldrich (Saint-Louis, Missouri, USA).

MTT stock solution was prepared in PBS at a final concentration of 5 mg/ml. Artesunate stock solutions (30 mg/ml) for permeation and MTT tests were prepared into a 5% (w/v) NaHCO_3_ aqueous solution followed by dilutions into adequate media. Artesunate for MTT assays was diluted into culture medium to final concentrations of 16, 12, 6, 3, 1.5, and 0.75 µg/ml. The donor for artesunate permeation studies was either a 0.75 µg/ml artesunate solution or a 20 µg/mg powder mixture of artesunate and corn-starch. Corn-starch is an inert excipient that was used to dilute the artesunate content in the powder formulation, increase the weight of the formulation, and thus allow its reproducible weighing for the permeation study [35, 36]. The donor solution for NaF permeation tests was prepared by dissolving NaF into KRB (25 µg/ml). All molecules’ solutions were filtered before use (0.22 μm filter).

### Artesunate formulations

Artesunate powder and solution formulations were prepared for the permeation studies.

For the powder formulation, pure artesunate powder and corn-starch were successively weighted in a plastic flask to a final concentration of 20 µg/mg artesunate in corn starch. The powder mixture was homogenized by shaking and the flask was stored at room temperature and protected from light until use. For the solution formulation, 30 mg of pure artesunate powder was weighted in a tinted glass vial and dissolved into 1 ml of 5% NaHCO_3_ (W/V) aqueous solution. The resulting solution was diluted in 2 ml of NaCl 0,9% (V/V) and then further diluted in KRB to a final artesunate concentration of 0.75 µg/ml. The solution formulation was prepared extemporaneously immediately before use.

### Cell culture

Nasal epithelial cells RPMI 2650 were used between passage 11 and 25 for all experiments. They were routinely cultured into 25 cm² polystyrene cell-culture flasks under standard conditions (humid atmosphere at 37 °C and 5% CO_2_). For cell passaging, cells were washed with PBS at 37 °C and then detached with 0.25% (w/v) trypsin-EDTA at 37 °C seven days after previous seeding. Cell viability was assessed using a standard trypan blue staining procedure. After counting, cells were seeded in a new flask at a $4 \times {10^4}{\text{cells}}/{\text{cm}}^{2}$ seeding density. Culture medium was changed every two to three days. Multilayer cell culture was performed into tissue-culture inserts according to the protocol previously described by Reich and Becker [37]. Briefly, $2 \times {10^5}{\rm{cells}}/{\rm{cm}}^{2}$ were seeded on permeable ThinCert® insert membranes and cultured under liquid-covered conditions (LCC) for eight days. After this period, the inserts were lifted at the air-liquid interface (ALI) and cultured for 14 more days to allow the formation of multiple cell layers and tights junctions [37, 38]. Transepithelial electrical resistance (TEER) was measured every two to three days during ALI culture. Experiments were performed in triplicate during at least three independent assays.

### Transepithelial electrical resistance (TEER)

**Fig. 1:**
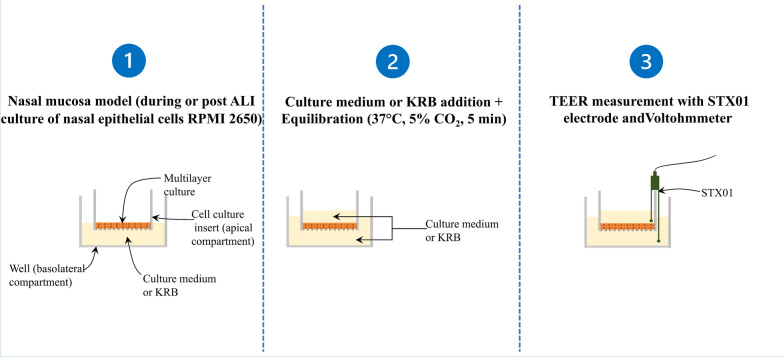
TEER measurement protocol. * ALI* air-liquid interface culture, *KRB* krebs-ringer buffer, *min* minutes, *TEER* Transepithelial electrical resistance

TEER was measured using a Millicell® ERS-2 Voltohmmeter (Merck, Darmstadt, Germany) and the STX01 chopstick electrode according to the manufacturer’s instructions (Fig. 1). Briefly, culture medium or KRB was added to the apical and basolateral compartments of the cell culture to final volumes of 1 ml and 1.5 ml, respectively. Cultures were then left to equilibrate at 37 °C for 30 min before measurements. TEER readings were corrected by subtracting blank filters values and normalized to the surface area of the membrane (1.13 cm²). Cell cultures having TEER values of at least 60 Ω.cm² were used for permeation assays [39].

### MTT cytotoxicity assay

A MTT cytotoxicity assay was performed to assess artesunate cytotoxicity on epithelial cells RPMI 2650 [40, 41]. Briefly, the cells were seeded at a density of $1.5 \times {10^4}{\text{ cells}}/{\text{cm}}^{2}$ in 96-well plates. After 24 h, artesunate drug dilutions were added and cells were incubated for 24 h. Following drug exposure, cells were gently washed twice with 100 µl PBS at 37 °C, before adding 110 µl of MTT stock solution diluted in culture medium (final MTT concentration : ~ 0.5 mg/ml). After four hours of incubation, the MTT solution was discarded and 200 µl of DMSO was added to each well. After one hour of incubation, absorbances were read at 485 nm using a Tristar2 LB 942 Multimode Microplate Reader (Berthold Technologies, Germany). Negative (cells without xenobiotics) and positive (cells with 25% v/v DMSO) controls were included. Results were expressed as cell viability (%) relative to negative control (100% viability). Acording to ISO 10993-5:2009, artesunate dilutions with cell viability percentages above 80% were considered as non-cytotoxic [42, 43]. Experiments were performed in triplicate during three independent assays.

### In vitro permeation studies

The permeation of the sodium salt of fluorescein (NaF) was used to validate the human nasal mucosa model derived from the culture of nasal epithelial cells RPMI 2650. Before the permeation test, multilayer cultures were rinsed with preheated KRB (37 °C) and their TEER were measured. In another 12-Well plate, 0.5 ml of donor solution was added into the donor chamber and 1.5 ml of KRB (37 °C) in the receptor chamber. The plates were incubated for 1 h under orbital shaking (37 °C, 5% CO_2_, humid atmosphere, 100 rpm). Samples (100 µl) were collected from the receptor chamber at fixed time intervals and immediately replaced with fresh KRB (37 °C). Permeated NaF was quantified into each sample using a Tristar2 LB 942 Multimode Microplate Reader (Berthold Technologies, Germany) with excitation and emission wavelengths of 485 and 535, respectively. Experiments were performed with five replicates during two independent assays. The apparent permeability coefficient of NaF (P_app_) was calculated with the following FDA approved equation and expressed in cm/s [39]:

$${P}_{app}=\left(\frac{V\times {C}_{f}}{{C}_{i}\times A\times t}\right)$$

V: volume of the acceptor chamber (cm^3^); C_f_ : drug concentration in the acceptor chamber at the end of the experiment (g/l or mol/l); C_i_: initial drug concentration in the donor chamber (g/l or mol/l); A: surface area of the membrane (cm²); t: duration of the experiment (s).

Artesunate permeation was evaluated in a similar way as described above for NaF. Aliquots of solution and powder formulations, equivalent to 0.357 and 200 µg of artesunate, respectively, were added into the donor chamber. Each permeation test lasted for 4 h and 200 µl samples were regularly collected from the acceptor chamber. TEER measurements were performed immediately before (TEER_i_), after (TEER_4 h_), and 24 h after (TEER_24 h_) test initiation. Artesunate permeation tests were performed in triplicate during three independent tests.

### Artesunate in vitro deposition study

**Fig. 2:**
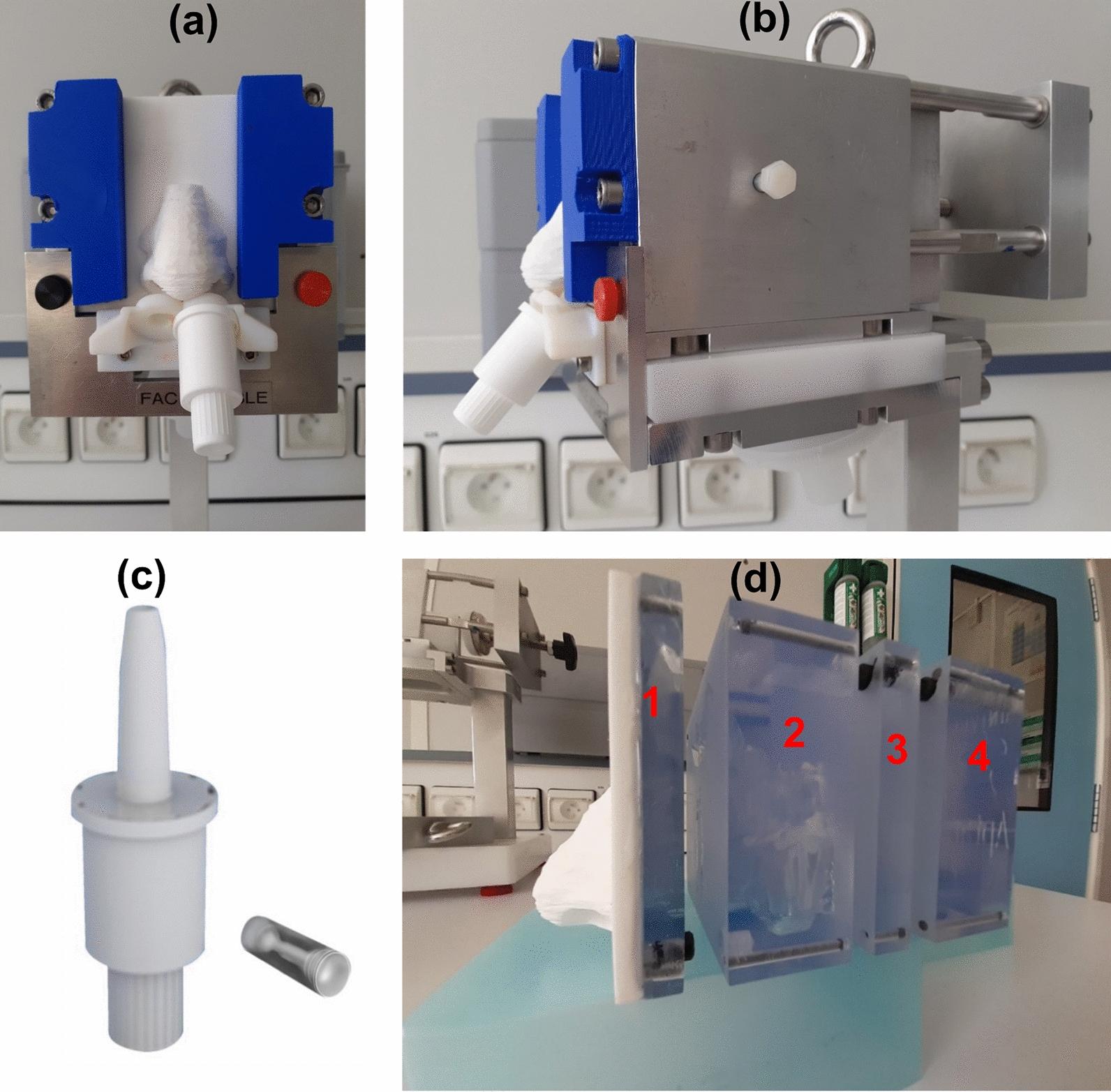
Nasal cast and spraying device (picture curtesy of Aptar pharma). **a**, **b** Pictures of the experimental set-up of artesunate powder deposition study. **c** Picture of the spraying device (UDSp). **d** Side picture of the nasal cast showing the nasal cavity divided in 4 blocks (in red): block 1 = nose, nasal valve, and frontal sinuses; block 2 = maxillary sinuses, frontal sinuses, nasal floor, turbinates, and ethmoids; block 3 = maxillary sinuses, sphenoids, nasal floor, ethmoids and turbinates; block 4: Sphenoids and nasopharynx

In vitro deposition of artesunate nasal powder was characterized in an adult male nasal cast (courtesy of Aptar/DTF/Univ. of Tours) with chemical quantification (Fig. 2). The nasal cast model used was designed from Computed Tomography images (CT scan) of a plastinated head model [44] and was previously validated as a predictive model for nasal aerosol deposition [45, 46].

**Fig. 3:**
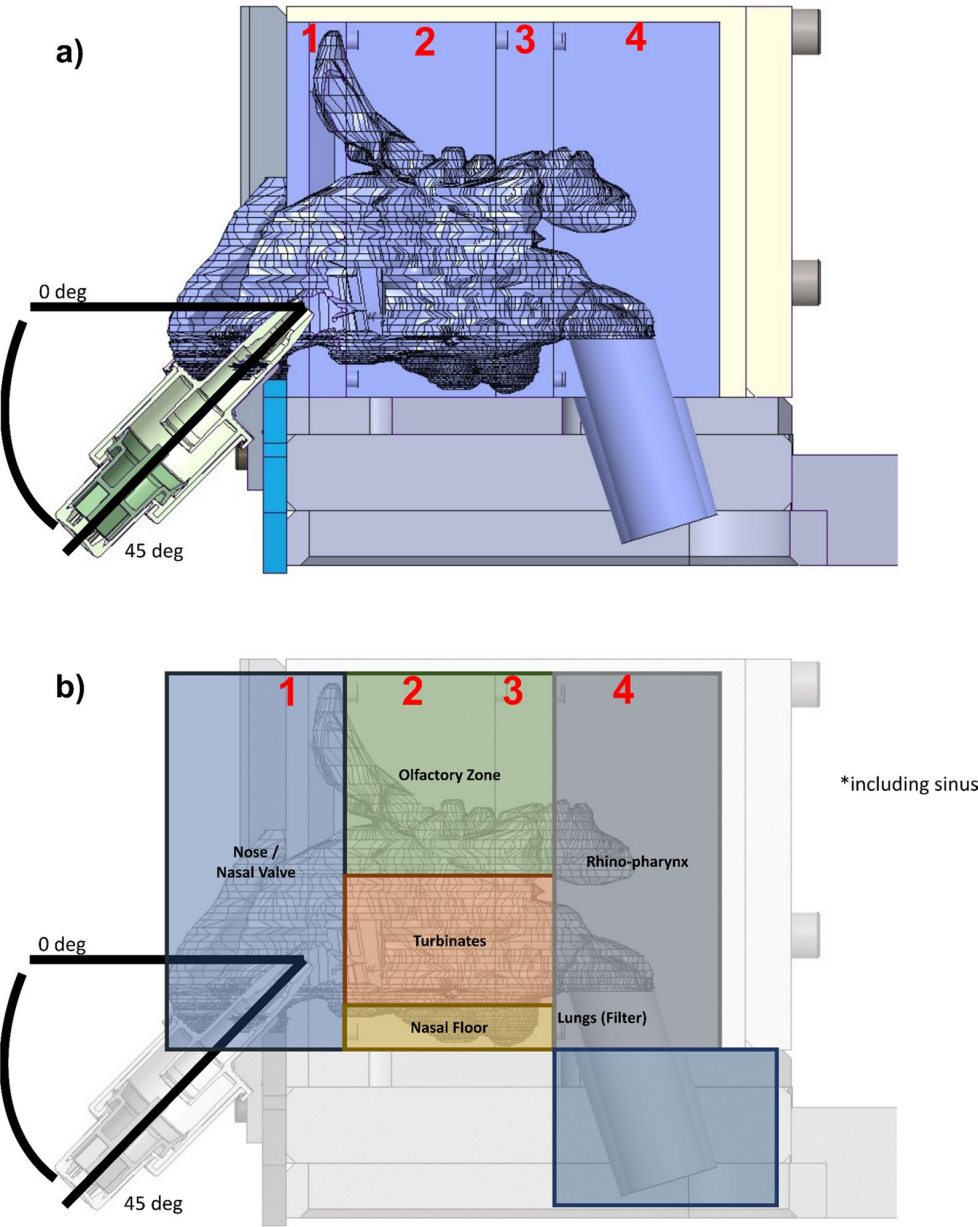
3D representation of the nasal cast and spray device with the nasal delivery place (picture curtesy of Aptar pharma). **a** 3D representation of the experimental set-up of artesunate drug deposition study. **b** 3D representation of the experimental set-up of artesunate drug deposition study with the blocks of the nasal cast and corresponding nasal regions. block 1 = nose, nasal valve, and frontal sinuses; block 2 = maxillary sinuses, frontal sinuses, nasal floor, turbinates, and ethmoids; block 3 = maxillary sinuses, sphenoids, nasal floor, ethmoids and turbinates; block 4: Sphenoids and nasopharynx

Six unidose (UDSp) devices supplied by Aptar Pharma (Le Vaudreuil, France) were filled with 10 ± 0.5 mg of artesunate powder. One device per nostril was manually actuated into the nasal cast with a fixed insertion depth of 1.5 cm, delivery angle (horizontal plane) of 45° and angle from the centre wall of 4°, after being humidified for 10 min at 3 lpm with a AMGH nebuliser (DTF, Saint-Etienne, France) (Fig. 3). The delivered dose was assessed by weighing each device before and after testing. The different regions of interest (Nose, Olfactory Zone, Turbinates, Nasal Floor, Nasopharynx and Lungs) were rinsed with a PBS solution at pH 8 and frozen at -80 °C to ensure artesunate remained stable.

### Analytical methods

For the permeation study, artesunate quantification was performed with an ultra-high performance liquid chromatography system (AcquityTM, Waters, Massachusetts, USA) coupled to a tandem mass spectrometer (XEVO-TQ-MS, Waters). Chromatographic separation was performed using an Ethylene Bridged Hybrid (BEH) C18 column (1.7 μm, 2.1 × 100 mm, Waters). The mobile phase consisted of 10 mM ammonium formiate in water (pH = 3) and 0.1% (v/v) formic acid in acetonitrile. Calibrators were prepared in KRB at concentrations ranging from 0.5 to 25 000 ng/ml. Before quantification, 20 µL of acetonitrile containing artesunate-d_4_ internal standard (at a concentration of 2.5 µg/mL) were added to 180 µL of each sample.

For the nasal deposition study, the samples were, firstly, thawed in a water bath at room temperature (20 °C). One millilitre (5 × 200 µl) of each sample was deposited on five filter membranes mounted on hydrophobic supports (OmniporeTM 10.0 μm, 47 mm). The membranes were thereafter left to dry overnight at room temperature. Artesunate was eluted from the membranes with 5 ml of methanol for each sample and the resulting solutions were analysed for drug determination. Artesunate quantification was performed using an Ultra-High Performance Liquid Chromatography system (Ultimate 3000, Thermo Fischer Scientific, Massachusetts, USA) coupled to a quadrupole-time-of-flight mass spectrometer (Impact II, Bruker, Massachusetts, USA). Chromatographic separation was performed using a Luna Omega Polar C18 column (1.6 μm, 2.1 × 50 mm, Phenomenex, California, USA). The mobile phase consisted of solvent (A), 1 mM ammonium acetate in ultra-pure water and 0.05% (v/v) acetic acid and solvent (B) 1 mM ammonium acetate in methanol and 0.05% (v/v) acetic acid. The lower limit of quantification (LOQ) of artesunate was set at 0.05 µg/g of solution. The drug deposition in each region of the nasal cast was expressed as the fraction (%) of the actual dose (100%) delivered with the device.

### Statistical analysis

All data management was performed with Microsoft Office Excel and GraphPad Prism 9.2.0 and 9.4.1 software. Statistical analyses, including Kruskal-Wallis test with Dunn’s multiples comparison test and Mann-Whitney test were performed with the significance threshold of α = 0.05. Data are presented as the mean ± standard deviation (SD) of at least two replicates.

## Results

### Cohesion of the human nasal mucosal model for in vitro permeation tests

**Fig. 4:**
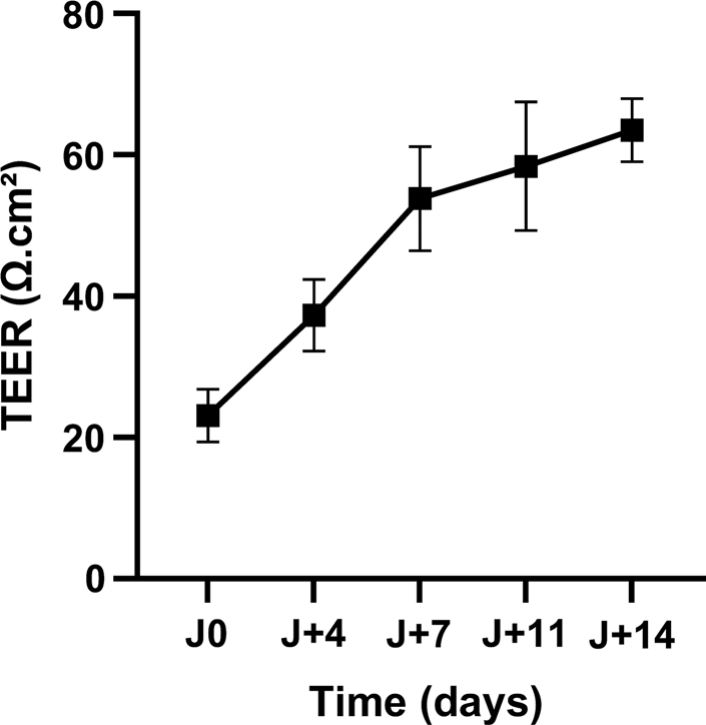
TEER during ALI culture of RPMI 2650 cells. TEER was measured on day 0, 4, 7, 11, and 14 during ALI culture of RMPI 2650 cells to monitor the formation of tight junctions. TEER values progressively increased and reached a mean value of 63 ± 4 Ω.cm² on day 14. Data are presented as the mean ± SD (n = 9). * TEER* Transepithelial Electrical Resistance, *ALI* Air-Liquide Interface

RPMI 2650 nasal epithelial cells were successively cultured under LCC then ALI conditions for 8 days and 14 days, respectively, to obtain appropriate cultures for in vitro drugs permeation tests. Monitoring of TEER values during ALI culture and determination of the P_app_ of NaF, a low weight hydrophilic marker of paracellular permeation, were used for the validation of the culture model. Average TEER values increased from 23 ± 4 Ω.cm² to 63 ± 4 Ω.cm² from the beginning to the end of ALI culture and demonstrated the gradual formation of tight junctions (Fig. 4). The P_app_ of NaF was 1.79 ± 0.07 × 10^− 6^ cm/s.

#### Effect of artesunate on the viability of nasal epithelial cells RPMI 2650

**Fig. 5:**
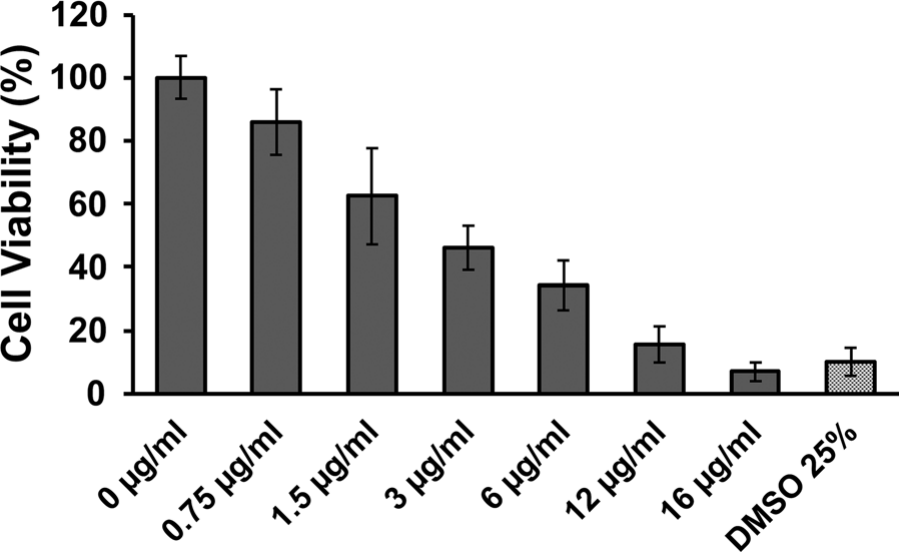
RPMI 2650 cell viabilities according to artesunate concentration using a MTT assay. Artesunate cytotoxicity was assessed on RPMI 2650 cells prior to performing permeation tests with AS solutions. No cytotoxicity was recorded for the 0.75 µg/ml artesunate solution (cell viability > 80%). Data are presented as the mean ± SD (n = 9). * DMSO* dimethyl sulfoxide

MTT assay was performed to determine the effect of artesunate on cultivated nasal cells viability. Cells were incubated with increasing artesunate concentrations for 24 h. Cell viability decreased from 86 ± 10% to 7 ± 3% upon treatment with artesunate concentrations ranging from 0.75 to 16 µg/ml (Fig. 5). Reduction in cell viability was significant when artesunate concentration was greater than 3 µg/ml (Kruskal-Wallis with Dunn’s multiple comparison, p-value ≤ 0,0049). The permeation tests using artesunate solution as donor were performed with a donor at a concentration of 0.75 µg/ml as it met the required non-cytotoxicity criterium (cell viability > 80%).

#### Artesunate permeation studies

**Fig. 6:**
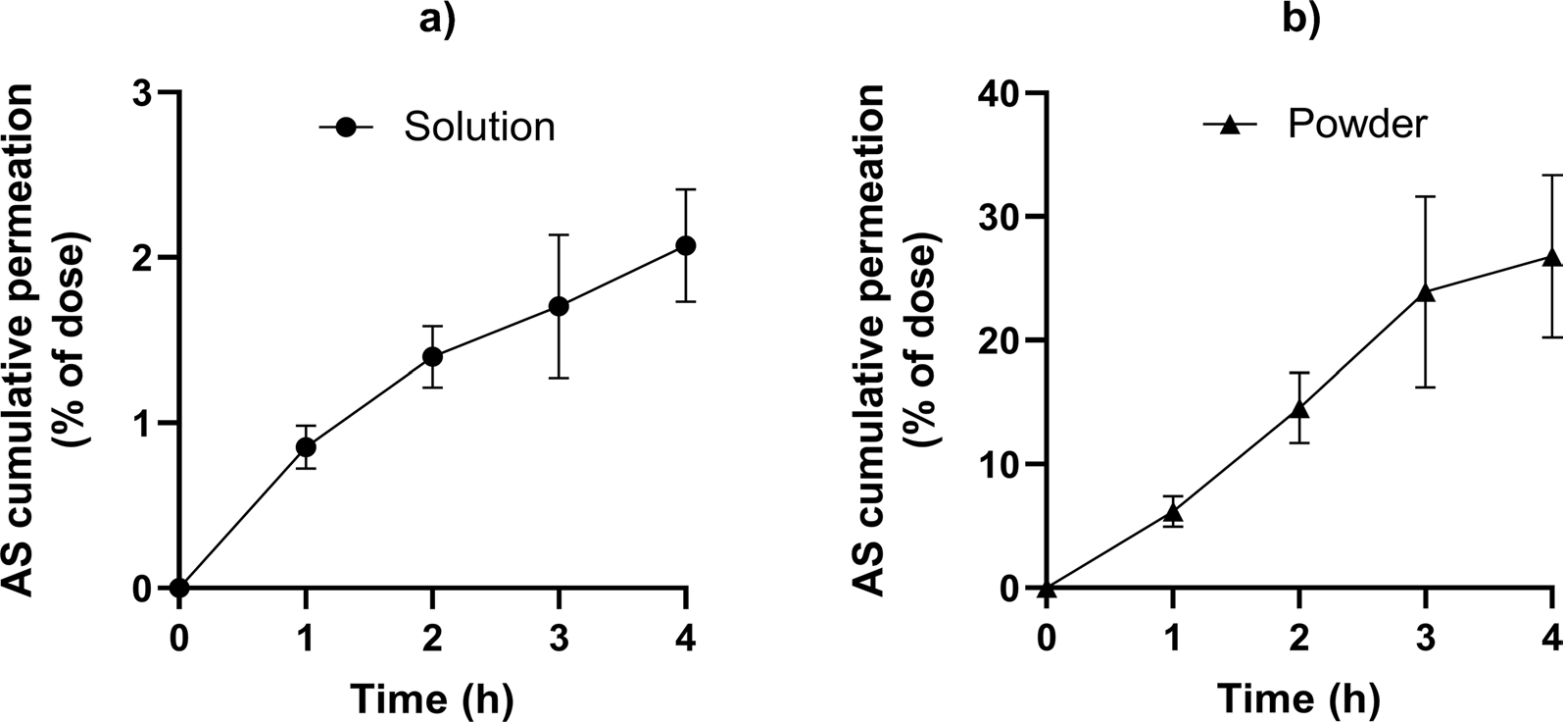
Artesunate permeation assay. **a** Permeation assay of artesunate in solution (0.75 µg/ml). **b** Permeation assay of artesunate in a powder (20 µg/mg). Artesunate permeation assays were performed on RPMI 2650 cell model using artesunate solution and powder formulations. Data are presented as the mean ± SD (n ≥ 3). * AS* artesunate, *min* minutes

The permeation profiles of artesunate on the human nasal mucosa model was assessed using a solution (0.75 µg/ml, artesunate stock solution diluted in KRB) or a powder formulation (artesunate powder diluted in corn starch, 20 µg/mg) (Fig. 6). The powder formulation demonstrated a better permeation efficiency (26.8 ± 6.6%) than the solution formulation (2.1 ± 0.3%) after four hours (p = 0.0003).

**Fig. 7:**
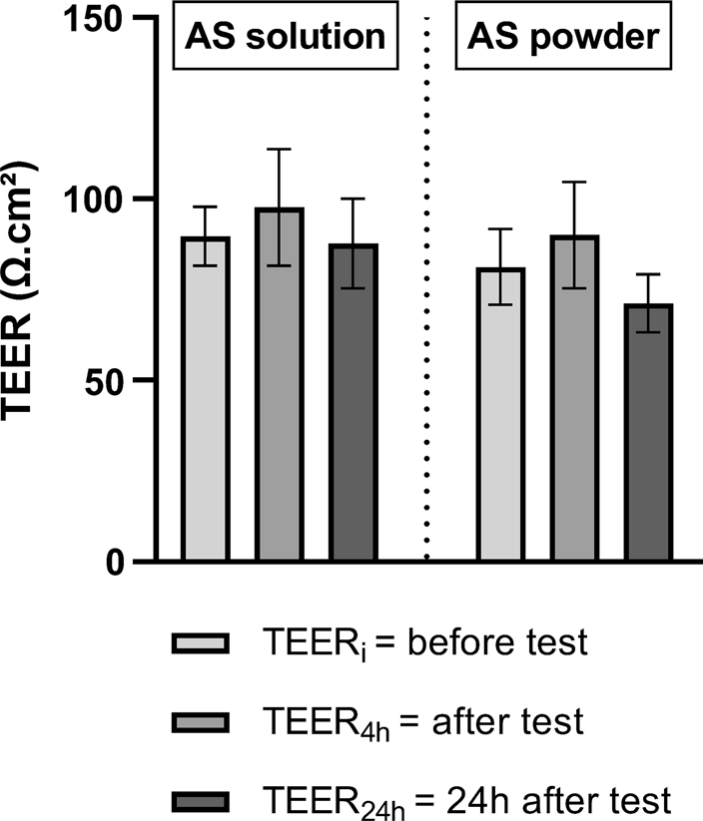
TEER measurements before and after the permeation assay of artesunate solution and powder formulations Cytotoxicity of artesunate was assessed according to TEER measurements before and after the permeation assay. Data are presented as the mean ± SD (n ≥ 3) * TEER* Transepithelial electrical resistance,* TEER*_*i*_ TEER before permeation assay,* TEER*_*4 h*_ TEER immediately after permeation assay,* TEER*_*24 h*_ TEER 24 h after permeation assay initiation

The TEER values before and after the permeation tests were not significantly different (Fig. 7). Using solution of artesunate, TEER values were 90 ± 8 Ω.cm², 98 ± 16 Ω.cm² (p = 0.3740), and 88 ± 12 Ω.cm² (p = 0.8791) before, immediately after and 24 h after the tests, respectively. Using artesunate powder, values were 81 ± 10 Ω.cm², 90 ± 15 Ω.cm² (p = 0.2859 ), and 71 ± 8 Ω.cm² (p = 0.0882).

#### Artesunate nasal deposition study

**Fig. 8:**
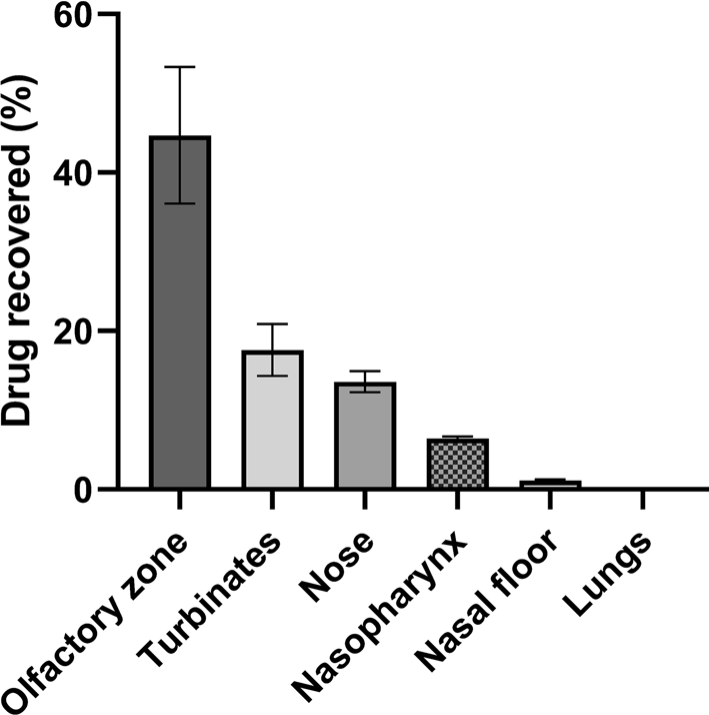
Distribution of artesunate powder by regions of the nasal cast (nasal cast use courtesy of Aptar/DTF/Univ. of Tours). Deposition experimentations were performed three times. The mean amount of drug sprayed into each nostril of the nasal cast model was calculated to be 9.8 ± 0.4 mg. Data are presented as the mean ± SD (n = 2)

Pure artesunate powder was filled into 6 UDSp devices and sprayed into each nostril of a human nasal cast model to assess the distribution pattern of the drug. The delivered doses assessed by weighing each device were correct with four devices (9.5 to 10.3 mg), but it exceeded the target dose (10 ± 0.5 mg) with the two last devices (11.6 and 11.9 mg) which were excluded from further analysis. The final amount of artesunate sprayed into each nostril was 9.8 ± 0.4 mg. The two nostrils of the cast were sprayed successively and the total artesunate deposition was measured from the full cast. Interestingly, the olfactory zone received the highest proportion of the sprayed artesunate (44.7 ± 8.6%) as expected, and the respiratory zone of the turbinates received 17.6 ± 3.3% of the dose (Fig. 8). For the other regions of the cast, namely, the nose, the nasal floor, the nasopharynx and, the lungs, the percentages of drug deposition were 13.6 ± 1.3%, 1.1 ± 0.2%, 6.4 ± 0.3% and, 0.11 ± 0.02%, respectively. The total dose recovery of artesunate amounted to 83.5 ± 6.2% of the sprayed dose.

## Discussion

The objectives of this preclinical proof-of-concept study were to investigate the capacity of artesunate powder to cross the nasal multilayer epithelial cells without local toxicity, and then to reach the olfactory and respiratory zones of the nose after being sprayed in the nostrils.

To that end, an in vitro model of human nasal mucosa was implemented using a two steps culture of nasal epithelial cells, first under liquid-covered conditions (LCC) for eight days in inserts, then at the air-liquid interface (ALI) for 14 more days to allow the formation of multiple cell layers and tights junctions.

This nasal mucosa model was previously proven to mimic the properties of human excised nasal tissue [47]. Indeed, TEER values, which measure tissue integrity and tightness, were demonstrated to be similar to that of the human nasal mucosa ex vivo (75–180 Ω.cm²) [48–51]. Moreover, the distribution of adherent and tight junction proteins, including E-cadherin and zonula occludens-1, as well as the expression of ABC efflux pumps, were shown to be similar to a leaky epithelium [39, 52]. Other studies demonstrated the formation of a mucoid material on the apical surface of the cells during ALI culture [38, 53].

After implementation of the in vitro model of human nasal mucosa, the accuracy of the nasal mucosa model by assessing its barrier function using TEER was confirmed. The TEER values obtained at the end of ALI culture were similar to those previously reported (66 to 79 ± 5 Ω.cm²) [38, 52]. Moreover, the average apparent permeability coefficient of NaF was in agreement with data reported from the same model [37] and from excised human nasal mucosa (3.12 $\times$ 10^− 6^ ± 1.99 $\times$ 10^− 6^ cm/s) [49]. Then, the agreement of these results with previous published data allowed us to establish the accuracy of the nasal epithelial cells RPMI 2650 culture.

After validation of this nasal cell model using TTER, artesunate permeation assays were performed with a solution and powder formulation of artesunate. The maximal non-toxic dose of artesunate in solution on nasal epithelial cells RPMI 2650 was assessed using a MTT assay after a 24-hour drug exposure. Artesunate was cytotoxic for concentrations greater than 0.75 µg/ml. One possible explanation for AS cytotoxicity was that the RPMI 2650 cell line originates from an anaplastic carcinoma of human nasal septum and that artesunate has an antitumoral activity on several in vitro models of cancer, including oesophageal, and lung cancers [52, 54–56]. Indeed, artesunate is readily hydrolysed in its active metabolite dihydroartemisinin (DHA) under aqueous conditions and the cleavage of DHA’s endoperoxide bridge is believed to induce an oxidative stress responsible for DNA damage, apoptosis, autophagy and ferroptosis [55, 57, 58]. In the light of these results, the permeation tests were performed with a 0.75 µg/ml artesunate solution as average cell viability for this concentration was above 80%. Moreover, considering artesunate potential cytotoxicity on the cells of the mucosa model, TEER was measured before and after both types of permeation assays to demonstrate that artesunate did not alter the integrity of the model during the permeation tests.

The permeation study showed that the powder formulation had a greater permeation efficiency than the solution (Mann-Withney test, p = 0.0003). This result may be explained by the higher drug concentration on the cell interface for the powder compared to the solution, as previously reported [38, 59, 60]. Indeed, the higher drug concentration gradient increases the concentration-dependant passive diffusion of the drug through inter- and intracellular pathways [59]. The hydrolysis of artesunate in DHA could have also contributed to the solution lower permeation efficiency by further reducing the concentration gradient of artesunate and its resulting passive diffusion through the cell layers. The greater permeation efficient of the powder formulation using a semi-quantitative biological test was previously demonstrated [61]. Beside artesunate anhydrous powder formulation offers a great advantage over the solution because it would prevent the chemical degradation of artesunate and increase the shelf life of the medication.

Considering artesunate in vitro permeation efficiency, it might be possible to optimise the diffusion of the drug by using permeation enhancing excipients such as chitosan [28]. Chitosan transiently opens the tight junctions between cells and increases the permeation of drugs through the paracellular pathway [24, 28]. Another alternative could be to explore other galenic formulations such as nanostructured lipid carriers (NLCs) [62].

The second objective was to investigate the areas of nasal deposition. The study demonstrated that a very significant amount of sprayed artesunate (45%) reached the olfactory zone allowing a nose-to-brain treatment that could be of major interest for targeting parasites sequestered in the cerebral microvasculature CM. Almost 20% of the sprayed dose reached the respiratory zone, providing also a systemic drug delivery avoiding the hepatic first-pass effect. Those results, combined with the demonstration of the substantial passage of artesunate powder through the nasal mucosa, emphasized the feasibility of an artesunate nasal drug delivery.

## Conclusion

This proof-of-concept study confirmed the success of intranasal artesunate treatment previously demonstrated during experimental cerebral malaria. Taken together, those results are part of the rationale for the use of intranasal artesunate as a malaria pre-referral treatment. The main advantage of this route compared to intrarectal is the nose-to-brain diffusion of artesunate which could avoid neurovascular impairment due to parasites sequestration. It is worth confirming this hypothesis by pre-clinical and clinical trials.

## Data Availability

The datasets used and/or analysed during the current study are available from the corresponding author on reasonable request.

## Abbreviations

ALI: Air-liquid interface
BEH: Ethylene Bridged Hybrid
DHA: Dihydroartemisinin
DMSO: Dimethyl sulfoxide
FBS: Foetal bovine serum
FDA: Food and Drug Administration
KRB: Krebs ringer buffer
LCC: Liquid-covered culture
MEM: Minimum essential medium
MS: Mass spectrometry
NaF: Fluorescein sodium salt
NLCs: Nanostructure lipid carriers
NEAA: Non-essential amino-acids
MTT: 3-(4,5-dimethylthiazol-2-yl)-2,5-diphenyltetrazolium bromide
PBS: Phosphate buffer solution
TEER: Transepithelial electrical resistance
